# Survey of topical exposure concerns for patients and caregivers dealing with atopic dermatitis

**DOI:** 10.3389/falgy.2023.1210973

**Published:** 2023-08-10

**Authors:** Grace Ratley, Ashleigh A. Sun, Korey Capozza, Kelly Barta, Ian A. Myles

**Affiliations:** ^1^ Epithelial Therapeutics Unit, National Institute of Allergy and Infectious Disease, National Institutes of Health, Bethesda, MD, United States; ^2^ Global Parents for Eczema Research, Santa Barbara, CA, United States; ^3^ Allergy and Asthma Network, Vienna, VA, United States

**Keywords:** atopic dermatatis, steroids, patient survey, triggers, eczema

## Abstract

**Background:**

Despite the recent expansion of treatment options in atopic dermatitis (AD), most management responsibilities fall on the patient and/or caregivers. Disease control often requires vigilance about and avoidance of common exposures, however the concerns for patients and caregivers living with AD have not been well enumerated.

**Methods:**

An IRB approved survey was distributed to the public to evaluate the patient and caregiver concerns for topical exposures and potential triggers.

**Results:**

323 people accessed the link to the survey with 259 providing response to at least one section of questions (response rate 80.2%). Results indicated that temperature and other weather related changes were the most common trigger. Nearly all respondents avoided at least one topical ingredient, with fragrances being the most common. Steroid exposure was common, however respondents expressed concerns about overall steroid exposure.

**Conclusions:**

Our results attempt to enumerate the daily topical exposure concerns for patients and caregivers living with AD. While our online survey is both limited and without mechanistic insights, our results provide insight to providers by highlighting the role of temperature in AD symptoms; identifying commonly perceived triggers; indicating the value of provider insight for topical product selection; and indicating that no specific aspect of topical corticosteroid exposure may alleviate the general steroid concerns for patients or caregivers.

## Introduction

Despite the recent expansion of treatment options in atopic dermatitis (AD) (1), most management responsibilities fall on the patient and/or caregivers. Disease control often requires vigilance about and avoidance of common exposures, including environmental triggers, skin care products, topical medications, as well as cleansers and detergents used in the home (2). However, patients' and caregivers' considerations and concerns about trigger avoidance have not been well enumerated (1).

## Materials and methods

### Survey

An IRB approved survey was distributed to the public through Research Match, Global Parents for Eczema Research, and the Coalition of Skin Diseases. 323 people accessed the link to the survey with 259 providing response to at least one section of questions (response rate 80.2%).

### Statistics

Body surface area (BSA) was calculated using SCORAD. Statistical analyses and visualization were conducted in R using the packages: ggplot2, epitools, pheatmap, sf, and corrplot.

## Results

**Figure 1:**
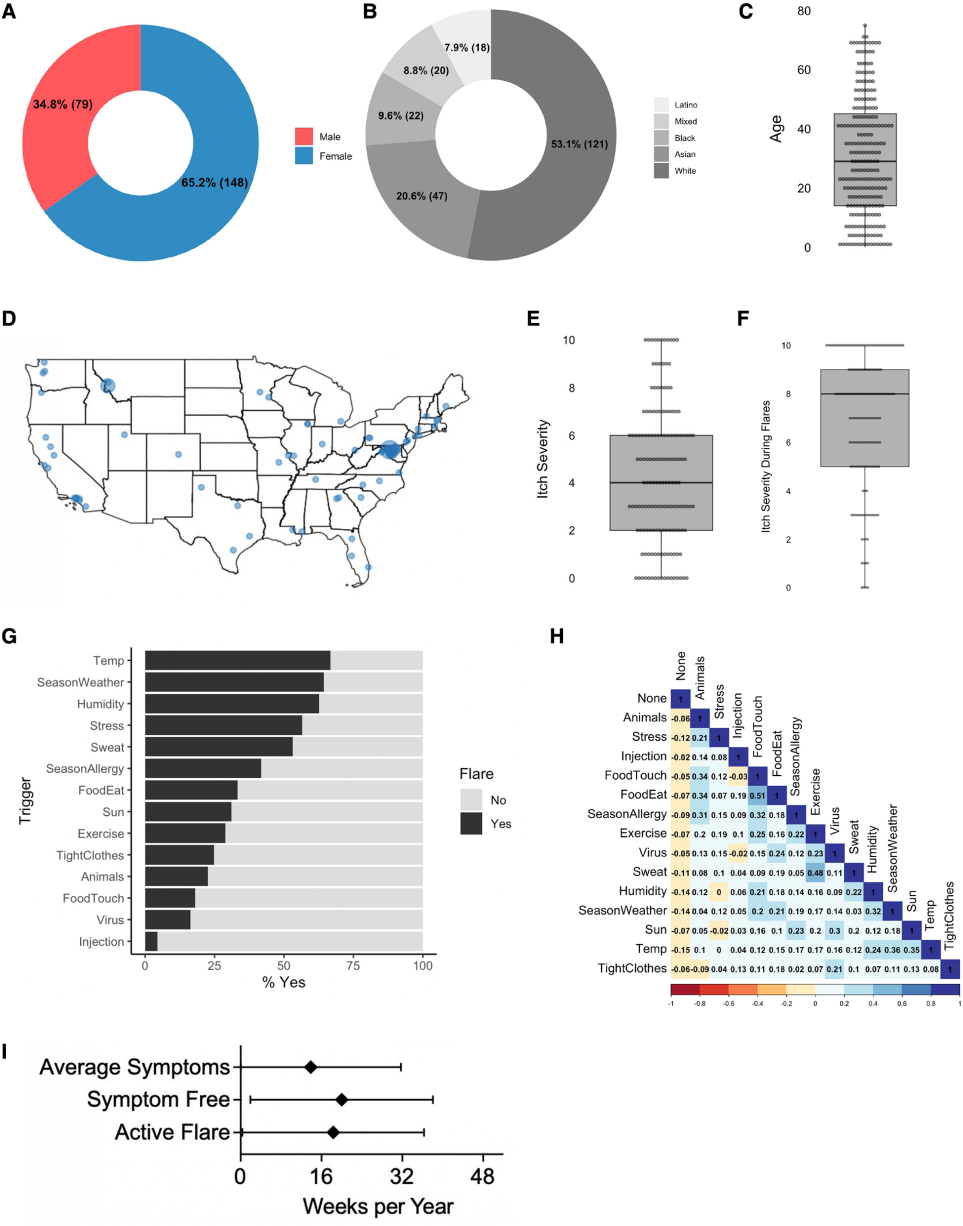
Demographics and triggers in respondents. (**A**) Self-identified gender (**A**, *N* = 227) and ethnicity (**B**, *N* = 228) of respondents. (**C**) Reponses to “how old is the person you are filling this out for?” (*N* = 237). (**D**) Zip codes for those identifying place of residence (*N* = 201). (**E,F**) Reponses to “how severe is your itch on an average day?” (**E**) or severity “during a flare?” (**F**) (*N* = 162). (**G**) % indicating the listed variable can induce a flare (*N* = 177). (**H**) Collinearity assessment for responses in G. (**I**) Mean ± SD of weeks per year the respondent indicated they or their child are in an active flare, symptom free, or are at baseline (*N* = 123). *N* values indicate the number of respondents that completed the indicated question.

Consistent with the patient and caregiver population, a female predominance was seen (shown in Figure 1A) (2). Our results reflected the US population but not the established racial disparities in AD (3) (shown in Figure 1B). The median age was 29 (shown in Figure 1C), mean age was 15.1 (standard deviation 13.6), and results extended across the US (shown in Figure 1D). Average itch severity was 4/10 (shown in Figure 1E) while during flares it was 8/10 (shown in Figure 1F). Temperature, seasonal weather, and humidity were the most common triggers (shown in Figure 1G) although the correlations between these similar factors were low (shown in Figure 1H). On average, respondents indicated being in an active flare 18.3 weeks of the year (shown in Figure 1I). For those indicating food as a potential trigger, “dairy” was the most common answer (*N* = 23) (shown in Figure 1G).

**Figure 2:**
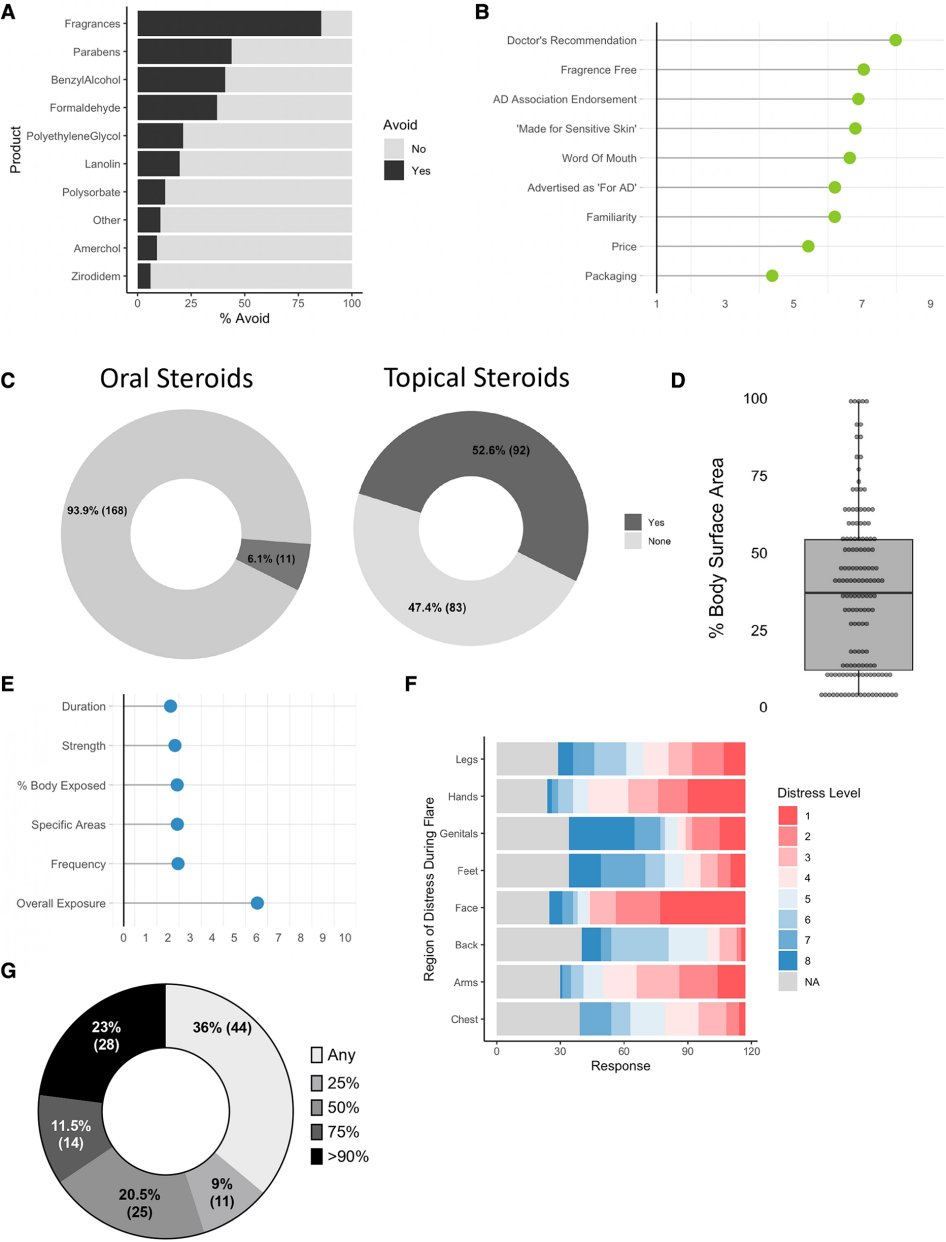
Enumerating concerns in patients and caregivers with AD. (**A**) For those affirming that “there are skin care products that you avoid because they worsen your or your child”s eczema”, % indicating the specified ingredient is avoided (*N* = 186). (**B**) Average rank score (1–9) for “most important factor in selecting a skin care product” (*N* = 182). (**C**) % of respondents indicating they have previously been prescribed oral (*N* = 179) or topical (*N* = 175) steroids for atopic dermatitis. (**D**) Participants were asked “when you apply steroids, which sites do you typically apply them to?”; derivation of body surface area (BSA) exposed to topical steroids (*N* = 144). (**E**) Mean response for level of concern (0–10) for overall and parameters of steroid exposure (*N* = 130). (**F**) Body site ranks for areas of most (1) to least (8) concern for application of steroids (*N* = 117). (**G**) Responses to “A new drug is announced that ONLY reduced the amount of topical steroids patients needed to use. How much of a reduction would make taking the new drug 'worth it'?” (*N* = 122). *N* values indicate the number of respondents that completed the indicated question.

The most avoided ingredient in topical cleaners were fragrances (35.0%) followed by parabens (18.0%) (Figure 2A). 5 respondents wrote in “sodium dodecyl sulfate”, which has been recently linked to allergic disease (4). A fake ingredient intended as a negative control (Zirodidem) ranked last with only 8 of 186 indicating avoidance (shown in Figure 2A). In total, 98.9% of respondents avoid at least one topical ingredient (*N* = 179). Physician recommendation was ranked as the most influential endorsement for topical products selection (shown in Figure 2B).

6.1% of respondents indicated they were currently taking oral steroids for AD (shown in Figure 2C). 52.6% indicated they used topical steroids (18.1% over the counter, 81.9% prescription). The median BSA coverage for topical corticosteroids (TCS) when used was 26.5% (shown in Figure 2D). When given a Likert scale for how concerned they were about TCS use, overall use ranked 6.5/10 with each of the specific aspects of use (such as potency, duration, or total coverage) scoring below 3 each (shown in Figure 2E). Concerns for exposure varied by body site with the face having the highest concerns of use (shown in Figure 2F). A plurality of patients (36%) reported that a novel drug might be “worth using” if it provided “any” reduction in steroid exposure (shown in Figure 2G). Some respondents indicated a concern for “topical steroid withdrawal”, an emerging but recently appreciated syndrome linked to discontinuation of TCS after prolonged use of mid-to-high potency formulations (5).

## Discussion/conclusion

Our results attempt to enumerate the daily topical exposure concerns for patients and caregivers living with AD. Our findings are limited by low *N* value and inablility to offer mechanistic insights. The reported avoidance of dairy (6), fragrances, and parabens (7) may reflect genuine allergic reactions or concerns without biologic ties. In addition, reports have shown that barrier function (as measured by transepidermal water loss) in nonlesional skin of patients treated with dupilumab also improves with EASI reduction (8). Thus, patients with worse symptom control may be more sensitive to exposures due to reduced barrier function. While our survey cannot separate these possibile mechanisms, our results do provide insights for providers to probe with their patients during clinical visits. The results are also limited by potential selection bias inherent to online surveys and the inability to obtain provider-level verification for the diagnosis and thus did not ask about infectious triggers of AD that have been reported, such as COVID-19 (9, 10). However, our results provide insight to providers by highlighting the role of temperature in AD symptoms; identifying commonly perceived triggers; and indicating the value of provider insight for topical product selection. The work also highlights the ability to use shared decision making in improving dermatologic conditions (11). While there are no specific aspects of TCS exposure which may alleviate the general steroid concerns for patients or caregivers, our results also indicate that oral steroids are used more than advised by the guidelines (1) and TCS are applied onto body surface areas above what has been indicated as safe (12).

## Data Availability

The original contributions presented in the study are included in the article/Supplementary Material, further inquiries can be directed to the corresponding author.
